# Predicting cervical intraepithelial neoplasia and determining the follow-up period in high-risk human papillomavirus patients

**DOI:** 10.3389/fonc.2023.1289030

**Published:** 2024-01-17

**Authors:** Ling Gong, Yingxuan Tang, Hua Xie, Lu Zhang, Yali Sun

**Affiliations:** ^1^ Department of Nursing, School of Nursing, Beihua University, Jilin, China; ^2^ Department of Computer Science and Technology, School of Computer Science, Northeast Electric Power University, Jilin, China; ^3^ Department of Gynecology, Jilin Central General Hospital, Jilin, China

**Keywords:** follow-up period, human papillomavirus, cervical intraepithelial neoplasia, prediction, genotype, cervical cancer

## Abstract

**Purpose:**

Despite strong efforts to promote human papillomavirus (HPV) vaccine and cervical cancer screening, cervical cancer remains a threat to women’s reproductive health. Some high-risk HPV types play a crucial role in the progression of cervical cancer and precancerous lesions. Therefore, HPV screening has become an important means to prevent, diagnose, and triage cervical cancer. This study aims to leverage artificial intelligence to predict individual risks of cervical intraepithelial neoplasia (CIN) in women with high-risk HPV infection and to recommend the appropriate triage strategy and follow-up period according to the risk level.

**Materials and methods:**

A total of 475 cases were collected in this study. The sources were from the Department of Gynecology and Obstetrics in a tertiary hospital, a case report on HPV from the PubMed website, and clinical data of cervical cancer patients from The Cancer Genome Atlas (TCGA) database. Through in-depth study of the interaction between high-risk HPV and its risk factors, the risk factor relationship diagram structure was constructed. A Classification of Lesion Stages (CLS) algorithm was designed to predict cervical lesion stages. The risk levels of patients were analyzed based on all risk factors, and follow-up periods were formulated for each risk level.

**Results:**

Our proposed CLS algorithm predicted the probability of occurrence of CIN3—the precancerous lesion stage of cervical cancer. This prediction was based on patients’ HPV-16 and -18 infection status, age, presence of persistent infection, and HPV type. Follow-up periods of 3–6 months, 6–12 months, and 3- to 5-year intervals were suggested for high-risk, medium-risk, and low-risk patients, respectively.

**Conclusion:**

A lesion prediction model was constructed to determine the probabilities of occurrence of CIN by analyzing individual data, such as patient lifestyle, physical assessments, and patient complaints, in order to identify high-risk patients. Furthermore, the potential implications of the calculated features were mined to devise prevention strategies.

## 1 Introduction

Global statistics on cancer in women indicate that cervical cancer ranked fourth in both incidence and mortality rate in 2012 (1). In 2020, there were over 604,000 cervical cancer cases and 341,000 deaths worldwide (2). The efficacy of vaccination and screening in preventing cervical cancer has been established, leading to increased awareness and participation in prevention programs among women. However, globally, the incidence and mortality rates of cervical cancer remain substantially higher in low-income and middle-income countries than in high-income countries; this is attributed to the lack of vaccination coverage, high-quality screening, timely treatment, and follow-up care services. A priority for public health managers worldwide is to take proactive measures to address the need for continuous and improved prevention and monitoring of cervical cancer. This aligns with the targets of the World Health Organization elimination initiative launched in 2020 to reduce cervical cancer incidence to below four cases per 100,000 women-years in every country (3). Furthermore, advancements in effective disease prediction and diagnosis are crucial for accurately identifying the target population.

Persistent high-risk HPV infection is recognized as the primary cause of CIN and cervical cancer. The pathogenesis of cervical cancer involves a prolonged period of development of precancerous lesions, such as the CIN1, CIN2, and CIN3 stages. The risk of developing invasive cervical cancer associated with CIN 1, CIN2, and CIN3 is 4 times, 14.5 times, and 46.5 times, respectively, higher than that of non-CIN. While most CIN 1 lesions resolve naturally, CIN2 and CIN3 incur the risk of malignant transformation (4–6). Studies, including randomized clinical trials, have indicated that HPV-based screening—characterized by high sensitivity and long-term negative predictive value—plays a significant role in primary screening methods, along with cervical cytology, in identifying potential cervical cancer cases and triage (7–9). Additionally, electronic colposcopy of the cervix and cervical biopsy are employed to determine the cervical lesion stage based on primary screening results. However, it is not advisable for all patients to directly undergo biopsy due to its associated low detection rate, wastage of medical resources, and invasive nature of biopsies. Therefore, accurate prediction of the risk of cervical lesions holds crucial clinical implications for early diagnosis and prevention of cervical cancer.

There are still some challenges in predicting cervical cancer, such as missing data in medical records and transient HPV infection. Poor data quality affects the accuracy of prediction. The uncertainty of the prediction model and the deficiencies in the data would lead to poor performance of the model during prediction and affect the reliability of the prediction results. In recent years, artificial intelligence (AI) has been gradually applied in the field of clinical medicine, especially in disease diagnosis and detection, for greater ability of learning and strong potentials in data processing (10–12). The application of AI is conducive to reducing the rate of missed diagnoses, saving more time, and improving accuracy for clinicians. AI technology has greatly improved the diagnostic accuracy of lung cancer and breast cancer through training CT and ultrasound images (13, 14). AI liquid-based cytology has resulted in efficient referrals to colposcopy, with higher specificity than manual screening methods (15). The Colposcopic Artificial Intelligence Auxiliary Diagnostic System has been explored to classify colposcopic impressions and suggest biopsies (16). AI technology can not only overcome the limitations of doctors’ subjective judgment and personal biases in diagnosis but also improve the accuracy of diagnosis and help to locate the lesion site (17–20). In the context of driving continuous progress in medical technology, there is an urgent need for an efficient and accurate method to determine the probabilities of occurrence of CIN through analysis of individual data such as information on lifestyle, physical assessments, and complaints so that high-risk patients can be identified and the potential implications of calculated features can be mined for further prevention strategies.

## 2 Materials and methods

### 2.1 Study design

**Figure 1:**
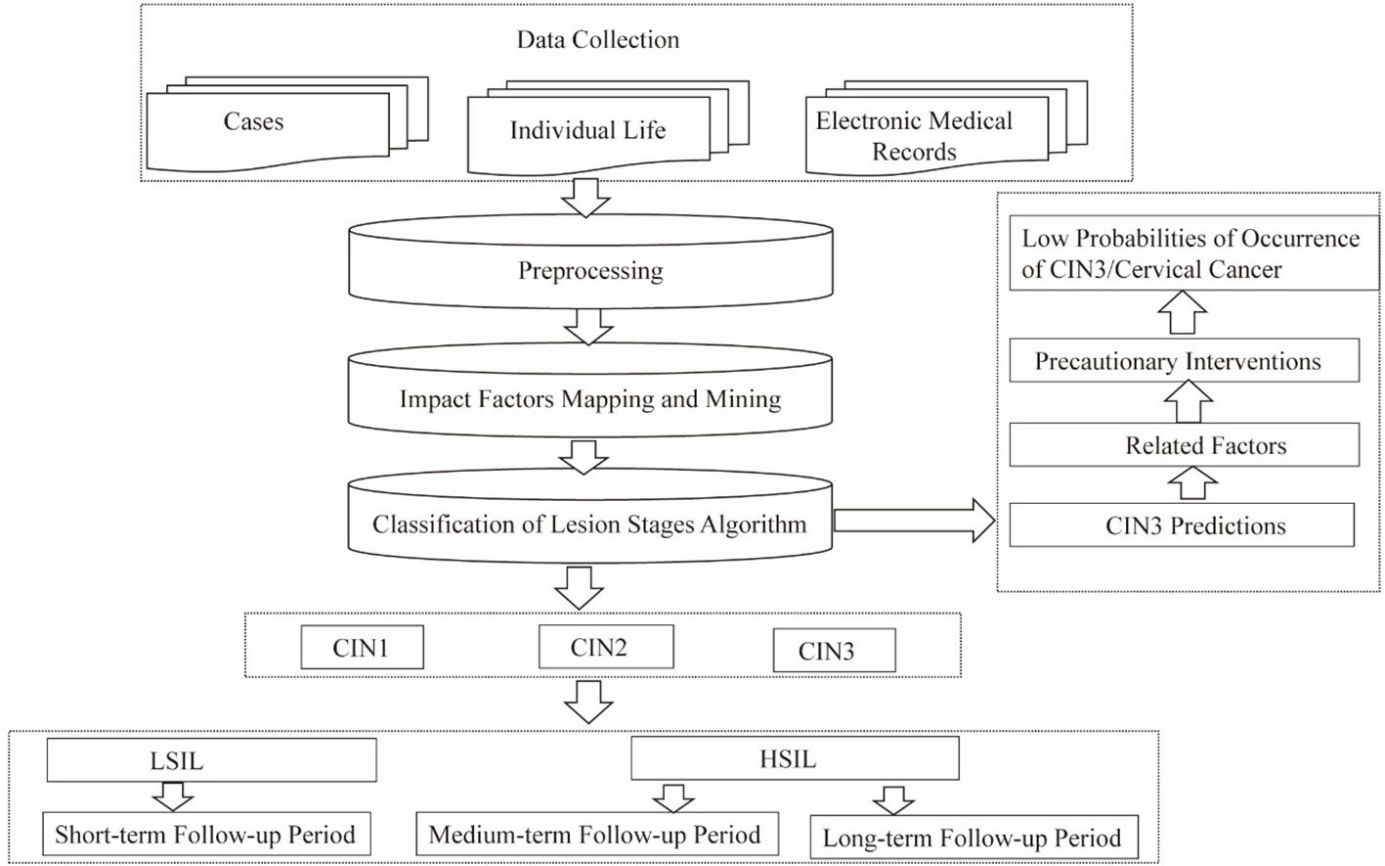
Mapping and mining of impact factors.

The pathogenesis of cervical cancer usually involves a long period during which precancerous lesions (such as CIN3) form, mainly caused by persistent infection with high-risk HPV. The aim of this research is to achieve early detection of the predisposing factors for precancerous lesions, based on high-risk HPV infection, and implementation of preventive patient interventions. Data preprocessing—including dataset construction and mapping and mining of impact factors, along with the CLS algorithm proposed in our research—enabled prediction of cervical lesions, exploration of predictive indicators, and risk classification of CIN. The findings yield valuable suggestions for the formulation of guidelines for patient follow-up periods at all levels and for advance implementation of preventive interventions, to effectively enable precise prevention strategies and reduce the probability of occurrence of cervical cancer (**Figure 1**).

### 2.2 Data preprocessing

#### 2.2.1 Datasets

The experimental environment is as follows: Python 3.7, Neo4j, and NetworkX 2.1 are configured under a Windows 10 operating system. Three Hadoop-distributed clusters of the CentOS 7 operating system were built, namely, HDFS, YARN, and Spark on YARN. A dataset constructing structure of the diagram for risk factors was collected by using crawler tools from the PubMed website, searching high-risk HPV, cervical cancer, HPV risk factor, and other similar terms, as literature retrieval words. The search yielded 2,221 pieces of medical literature.

The case data were collected mainly on the basis of cases with high-risk HPV infection and lesions, cases with high-risk HPV infection but no lesions, cases without high-risk HPV infection but lesions (i.e., cases that tested HPV-negative, but with lesions), and cases without high-risk HPV infection and no lesions.

A total of 475 cases were collected in this study. The sources were as follows: the Department of Gynecology and Obstetrics at Jilin Central General Hospital, case report articles about HPV on the PubMed website, and clinical data for cervical cancer in The Cancer Genome Atlas (TCGA) database.

#### 2.2.2 Mapping of key risk factors

The electronic medical record text, which is different from ordinary text, usually has a relatively complete structure, including patients’ personal information, main complaints, personal history, physical examination results, and auxiliary examination results, with little noise data. Examples of the style of entries include “the patient had vaginal bleeding one month ago,” “denied history of drug allergy,” and “denied familial inherited diseases”. Therefore, the set of keywords for patient case data can be obtained by natural language processing methods. Key words representing textual information were directly extracted—e.g., “vaginal bleeding” for “the patient had vaginal bleeding one month ago”—and numerical information was extracted according to the rules shown in **Table 1**.

**Table 1:** Extraction Rules for Abnormality of Risk Factors.

Risk Factors	Extraction Rules for Abnormality
Age	> 30 years
Age at Menarche	> 14 years or <12 years
Age at First Sexual Intercourse	< 18 years
Number of Sexual Partners	>2
Age at First Full-term Pregnancy	<18 years
Number of Vaginal Births	> 2
Number of Pregnancies	> 2

### 2.3 Method

#### 2.3.1 Classification of the lesion stage algorithm

The challenges involved in predicting lesion stage by machine learning methods involve determining what kind of data and what kind of features to analyze and calculate. The corresponding test values for patients are commonly used for training and analysis in machine learning methods, which poses great obstacles due to insufficient amount of data. The larger the amount of data and the more values available in machine learning, the more accurate the training is. However, there are many missing values and few positive samples when collecting data, which causes failures of application of many disease prediction models. Therefore, the mechanism of the disease should be fully considered when selecting features to enable more accurate prediction. The Classification of Lesion Stages (CLS) algorithm proposed in this study gives full consideration to the pathogenic mechanism and selects appropriate features for analysis, which has practical significance for the prediction of cervical lesions.

#### 2.3.2 Types of high-risk HPV and classification of lesion stages

There are more than 100 types of high-risk HPV; 16 common types, namely, HPV-16, -18, -58, -52, -31, -51, -33, -35, -56, -26, -39, -53, -66, -67, -70, and -45, were analyzed and used for the calculations in this study, as nodes in the structure of the risk factor graph and connected with many other factors.

Cervical lesions are divided into three grades: CIN1, CIN2, and CIN3. The CIN3 stage has a high probability of transformation into cervical cancer. In 2014, the World Health Organization reclassified it into low-grade squamous intraepithelial lesion (LSIL) and high-grade squamous intraepithelial lesion (HSIL), further simplifying the original classification. LSIL refers to the original CIN1 stage, and HSIL includes the original CIN2 and CIN3 stages. In this study, both classification methods were adopted in the analysis and calculation stage, which was conducive to more detailed analysis. We described the differences in neighbor risk factor nodes and neighbor HPV genotypes between CIN1, CIN2, and CIN3. The factors with node relation value greater than 3 were selected as close factors, among which differences were compared and the degree of difference was calculated.

#### 2.3.3 Prediction of lesion stages

Based on the set of key risk factors, we extracted the risk factors that were abnormal in case history and the HPV types with which the patient was infected by natural language processing. According to the principle of abnormal extraction of risk factors, we identified key risk factors for patients with abnormal p collection ${AF}_{p}{=\{af}_{1,}{af}_{2},\ldots \ldots ,{af}_{n}\}$ , including patients’ HPV types and their risk factors that were abnormal. The predictive value of a patient’s classification relative to CIN1 was calculated by the following Equation 1.


(1) $${CIN1}_{p}=\sum_{m=1}^{n}W_{\left(AF_{m},cin1\right)}$$

When the abnormal factors for a patient included those in *cin2Element* or *cin3Element*, it indicated that the patient had factors unique to CIN2 or CIN3. To describe this difference, the degree of difference was introduced to calculate the extent of difference of CIN2 or CIN3 relative to CIN1 in the current situation for each patient, using Equation 2. Abnormal factors as unique ones that appeared in CIN2 or CIN3 were remembered as ${CIN2ELE}_{p}=\{{cin2Ele}_{\left(p,1\right)},{cin2Ele}_{\left(p,2\right)},\ldots \ldots ,{cin2Ele}_{\left(p,n\right)},\}$ , ${CIN3ELE}_{p}=\{{cin3Ele}_{\left(p,1\right)},{cin3Ele}_{\left(p,2\right)},\ldots \ldots ,{cin3Ele}_{\left(p,n\right)},\}$ 。


(2) $${Diff}_{\left(p,\;cin2\right)}=\frac{\sum_{m=1}^{n}W_{\left(cin2Ele_{\left(p,m\right)},cin2\right)}}{n}$$

$W_{\left(cin2Ele_{\left(p,m\right)},cin2\right)}$ —connected edge weights of factor $\;of\;cin2Ele_{\left(p,m\right)}$ and CIN2 in risk factors—figure structure.

*n*— number of elements in ${CIN2ELE}_{p}$ ${Diff}_{\left(p,cin2\right)}$ —degree of difference in patients’ p between CIN2 and CIN1.

The degree of difference in patients with p between CIN3 and CIN1 ${Diff}_{\left(p,cin2\right)}$ was calculated in the same way.

The degree of difference calculation should be introduced into the classification predicted value of CIN2 or CIN3, which is calculated by Equations 3, 4.


(3) $${CIN2}_{p}={Diff}_{\left(p,\;cin2\right)}*\sum_{m=1}^{n}W_{\left(AF_{m},cin2\right)}$$

(4) $${CIN3}_{p}={Diff}_{\left(p,\;cin3\right)}*\sum_{m=1}^{n}W_{\left(AF_{m},cin3\right)}$$

From the above calculation, the three classification predictive values of patient “p” can be obtained. In order to more accurately determine which category the patient belongs to, the risk level of the patient is introduced into the analysis. Patients at a low-risk level—which means that their risk of infection with high-risk HPV is very low—have low possibility of cervical lesion. Therefore, we predict that patients at a low-risk level will be disease-free (CIN−). In high-risk patients, i.e., those with a high risk of infection with high-risk HPV, the likelihood of lesions is also high. The prediction result with the largest predictive value of the three-stage classification is selected as the final prediction result. If the maximum value is the predicted value FOR CIN2 or CIN3, it is classified as HSIL; if the maximum value is the predicted value FOR CIN1, it is classified as LSIL. For intermediate-risk patients, this analysis is somewhat difficult, because for these patients, the risk level value is around 0.5, which represents an almost risk of occurrence of HSIL or LSIL. Patients in this category require more cautious management. In order to reduce the rate of missed diagnoses rate, degree of difference analysis is conducted in the present study. If the value of the degree of difference of patients with CIN2/CIN3 is greater than 2 at any stage, it is identified as a large difference and directly classified as HSIL because the possibility of CIN2/CIN3 stage is stronger. If the value of degree of difference is not greater than 2 at either stage of CIN2/CIN3, the patient does not have a high stage difference. In this scenario, identification as CIN1 and classification as LSIL is more likely.

## 3 Results

### 3.1 Follow-up period

**Figure 2:**
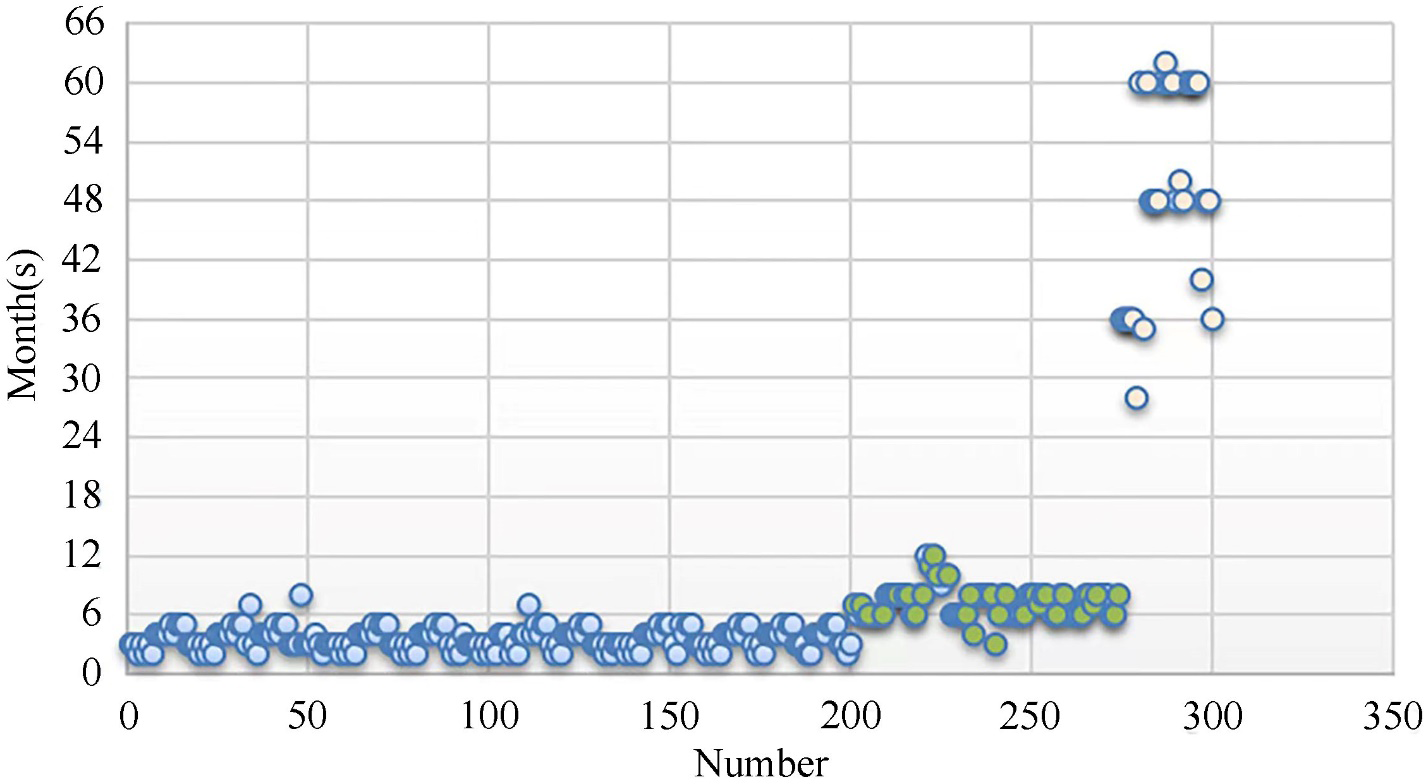
Follow-up periods for patients at the three risk levels (Blue: Low-risk Level, Green: Medium-risk Level, Beige: High-risk Level).

After calculating risk levels for all patients, follow-up periods for patients at different risk levels were statistically analyzed (**Figure 2**). Each blue circle represents the suggested follow-up period for a patient at a high level of risk, green ones show follow-up periods for patients at medium risk, and beige ones represent suggested follow-up periods for patients at low risk. There was a clustering of data at different levels.

After summarizing the data for the above groups, the follow-up periods for patients at different risk levels were obtained. Respectively, for high-risk, medium-risk, and low-risk patients, follow-up at 3 to 6 months, 6 to 12 months, and 3 to 5 year intervals was suggested (**Table 2**).

**Table 2:** Follow-up Periods for Patients of Different Risk Levels.

Risk Level	Follow-up Period
Low-Risk	3-5 years
Medium-Risk	6-12 months
High-Risk	3-6 months

### 3.2 Prediction of CIN

The 16 types of high-risk HPV, CIN1, CIN2, and CIN3 exist in the risk factor graph structure as nodes connected with many other factors, and the weight of the edge represents the closeness between them and the risk factors. The neighbor nodes in the graph structure were used to observe the relevant factors for different disease stages and the nodes’ characteristics. The names and edge weights of key risk factors and high-risk HPV types that were directly related to CIN1, CIN2, and CIN3 were the output. CIN3 node’s top-5 neighbor nodes and their relationship values are addressed as below (**Table 3**).

**Table 3:** CIN3 Node’s Top 5 Neighbor Nodes.

Neighbor Nodes	Value of the Relationship with CIN3 Weight
HPV 16	3.004129552
HPV 18	3.00267266
Age	3.001884209
Persistent HPV Infection	3.001449165
HPV Type	3.000758473

The risk classification of each patient warranted consideration. In addition, for lesions at different stages, their close risk factor neighbor nodes and the relationship value was different. Consideration of the difference of factors at different stages was conducive to better classification and prediction of patients’ lesions.

The values of the relationship with the CIN3 weight of all the top-5 neighbor nodes were between 3 and 3.005, which means that the factors were reliable predictive parameters for CIN3; in order, they were HPV-16, HPV-18, age, persistent HPV infection, and HPV type. HPV type and infection represent four of the five closest neighbor nodes of CIN3. Obviously, factors closely related to HPV made a large contribution to precancerous progression. The top two factors were the two high-risk genotypes 16 and 18, in line with current studies that consider them the predominant causes of precancer or cervical squamous cell carcinoma. In recent years, extant works have yielded similar results as our study: HPV subtypes in different age groups and different regions have different characteristics, according to epidemiological statistical data. Moreover, the differences also reflect the different levels of cervical lesions (21).

When analyzing the CLS and identifying related risk factors and high-risk HPV types, we found that different lesion stages had different correlations with high-risk HPV types. Three genotypes, mentioned in **Table 4**, describe CIN1-related high-risk HPV and relation value with CIN1. In descending order of risk, they are HPV-18, -16, and -45. The top two values are above 3.0, which is remarkably higher than the value for HPV 45. It is suggested that HPV 18 has the closest relationship with CIN1 and HPV 45 takes the third place with a relatively low value.

**Table 4:** CIN1 Relevant High-risk HPV and Value.

Type of High-risk HPV	Relation Value with CIN1
HPV 18	3.001518086
HPV 16	3.000897878
HPV 45	1.000011528

As shown in **Table 5**, CIN2-related high-risk HPV genotypes include those found in CIN1 as well as HPV 31. HPV 16 is the primary type. The relation values with CIN2 for HPV-16 and -18 are greater than 3.0, although the values for HPV-31 and -45 are just over 1.0. Evidently, HPV-16 and -18 are predominant factors leading to CIN2 among high-risk HPV genotypes. Despite the values for the other genotypes not being as high, HPV-31 and -45 emerge, among many other genotypes, as CIN2-relevant high-risk HPV genotypes.

**Table 5:** CIN2 Relevant High-risk HPV and Value.

Type of High-risk HPV	Relation Value with CIN2
HPV 16	3.003927177
HPV 18	3.003502006
HPV 31	1.00005263
HPV 45	1.000007755

Calculations implicate 14 genotypes as causes of CIN3 from the perspective of high-risk HPV (**Table 6**). They can be divided into three echelons according to relation value with CIN3 ≥3.0, ≥2.0 and<3.0, and ≥1.0 and<2.0. In the first echelon, HPV-16 and -18 display the most intimate relationship with CIN3. HPV-58 and -31 appear in the second echelon and HPV-52, -56, -66, -51, -39, -35, -33, -45, and -26 emerge in the third echelon, in descending order.

**Table 6:** CIN3 Relevant High-risk HPV and Value.

Type of High-risk HPV	Relation Value with CIN3
HPV16	3.004129552
HPV 18	3.00267266
HPV 58	2.000134558
HPV 52	1.000043481
HPV 31	2.000129077
HPV 51	1.000015006
HPV 33	1.000014283
HPV 35	1.000014325
HPV 56	1.000015488
HPV 26	1.000007551
HPV 39	1.000014413
HPV 66	1.00001536
HPV 45	1.000007829

CIN3 was correlated with multiple high-risk HPV types; in other words, when these high-risk HPV types occur, there is a greater probability of development of CIN3. At the same time, we found that HPV-16 and -18 have a strong impact on each of the three stages. A number of studies over the years have also shown that these two HPV genotypes are associated with the highest risk of occurrence of lesions and even cervical cancer, and the three common types of cervical cancer vaccines inevitably cover these two genotypes. Compared with the CIN1 stage, it was found that the HPV31 genotype was a unique high-risk type for the CIN2 stage, indicating that upon infection with HPV31, the likelihood of development into the CIN2 stage is higher. High-risk HPV infection warrants more attention. It also indicates that multiple genotypes of infection leads to greater likelihood of high-grade lesions.

### 3.3 Experimental analysis

We introduced an experimental evaluation index and conducted evidence-based analysis based on the diagram structure of risk factors. Finally, classification to predict the cervical lesion stage of patients and experimental verification through a total of 125 collected case data, excluding the data for cases that have developed into cervical cancer, was carried out. Comparative experimental analysis between the CLS algorithm proposed in this study and SMOTE-LSTM (22) was conducted.

At a statistical level, the results of disease diagnosis are described in terms of sensitivity and specificity. Sensitivity refers to the ability of diagnostic tests to detect disease when people are sick, as shown in the calculation Equation 5. Specificity refers to the ability of diagnostic tests to exclude disease when people are not sick, as shown in the calculation Equation 6.

TP (true positive): The prediction corresponds to the number of people diagnosed with a certain stage of the disease.

FP (false positive): The prediction does not correspond to the number of people diagnosed with a certain stage of the disease.

FN (false negative): The prediction does not correspond to the number of people free from disease.

TN (true negative): The prediction corresponds to the number of people free from disease.


(5) $$sensitivity=\frac{TP}{TP+FN}$$

(6) $$specificity=\frac{TN}{TN+FP}$$

Sensitivity and specificity are often used to evaluate the authenticity of outpatient results. In order to evaluate the classification results of disease prediction more accurately, the definition of true positive in this study has been modified. In general, true positive indicates the condition of finding disease and predicting disease; that is, patients with the disease are correctly predicted to be patients with the disease. However, in the study, true positive is to predict not disease but accurate disease stage. These changes were made to improve the accuracy of CLS.

The CLS algorithm put forward in this research and the SMOTE-LSTM algorithm were compared based on the two aspects of specificity and sensitivity. Sensitivity represents the ability to identify patients, and specificity represents the ability to identify non-patients, i.e., the ability to be assessed as disease-free.

**Figure 3:**
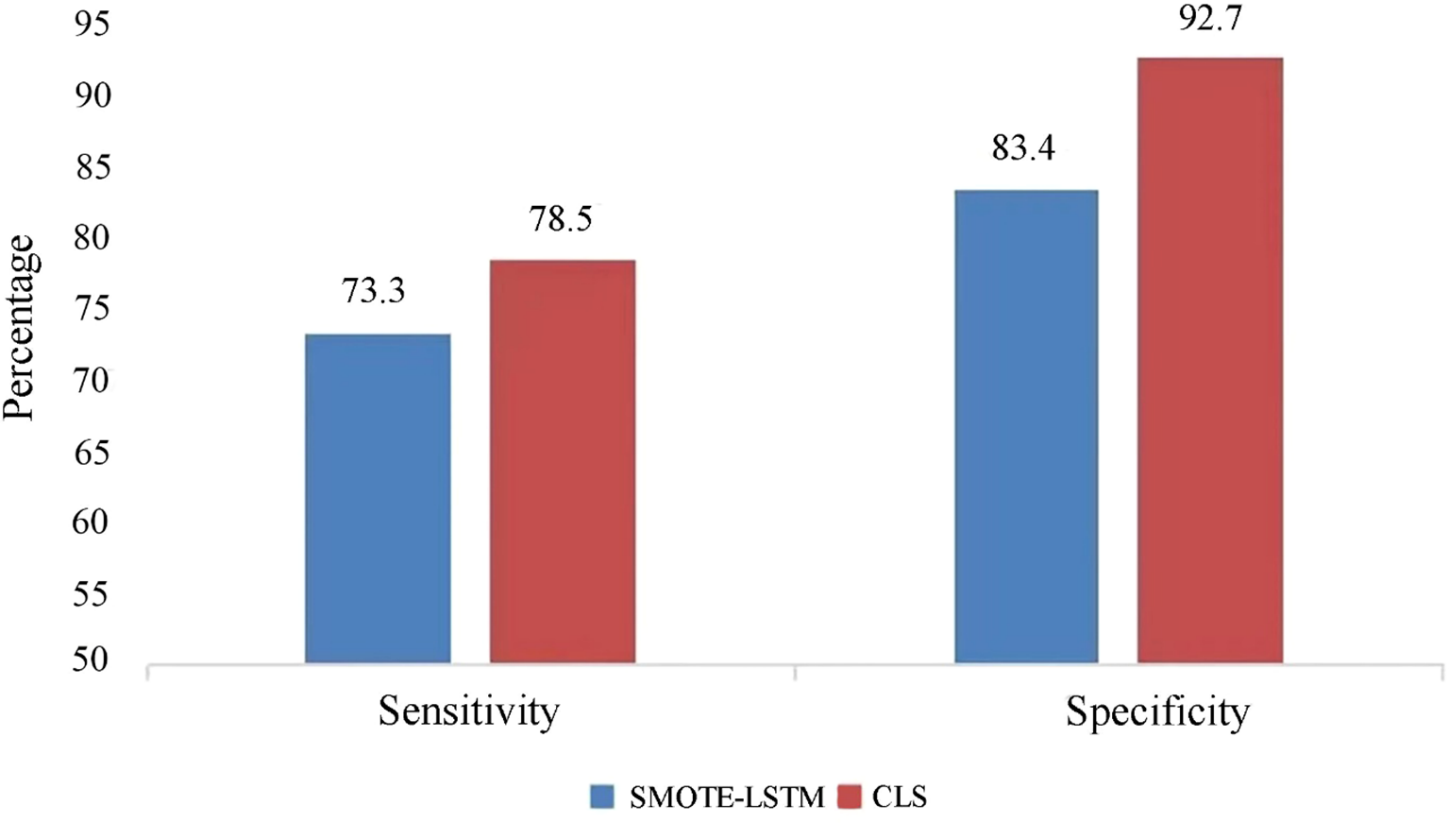
Comparison of sensitivity and specificity of prediction methods for cervical lesions.

The experimental results of lesion prediction are shown in **Figure 3**. It can be clearly seen that the sensitivity and specificity of the CLS algorithm proposed in this study are higher than those of the comparison algorithm. This is because we have fully considered the principle of disease application, that is, the relationship between high-risk HPV infection and cervical lesions. A comprehensive analysis of the infection risk level of the patients themselves was carried out, so as to avoid missed diagnoses of those patients who have not tested positive for high-risk HPV but do have lesions. Degree of difference analysis was introduced to analyze the differences between related risk factors at different disease stages, so as to classify and predict the disease stages of patients better. In addition, the specificity of the CLS algorithm proposed in this study reached 92.7%, which indicates that our algorithm shows good ability to distinguish non-patients from patients. The CLS algorithm is therefore a tool for medically assisted decision-making that can effectively reduce the occurrence of overexamination.

## 4 Discussion

Accurate decision-making regarding the appropriate follow-up period for a target population with high-risk HPV infection can be time-consuming and challenging for clinicians, given the multitude of factors to consider. Prolonging the follow-up period increases the risk of missing the occurrence of cervical lesions, potentially leading to missed diagnostic opportunities before lesions develop. If the follow-up period is set too short, it may result in excessive examination, wasting medical resources and posing harm to patients’ health. In 2020, the American Cancer Society updated its guidance to extend HPV screening intervals to 5 years based on accumulated evidence (23). However, disparities exist in recommendations from different academic organizations. According to the ATHENA trial, colposcopy is recommended if the patient tests positive for either HPV 16 or 18. Unfortunately, HPV testing can detect viral subtypes rather than persistent infection, which is an important factor in carcinogenesis. Girls and women tested positive for HPV subtypes -35, -39, -51, -56, -59, -66, or -68 are advised to undergo rescreening in 12 months (24). HPV infection genotype is important in detecting cancer and should be considered in triage management (25). To avoid excessive examinations and reduce the burden on patients, a more personalized diagnosis is recommended based on individual conditions. Physicians often manage patients according to their practical experience, acquired knowledge, guidance from predecessors, or research reports in journals, mainly relying on their subjective judgment. Evidence-based medicine not only focuses on doctors’ clinical experience but also emphasizes the use of scientific evidence to guide clinical practice. Computers, as tools for data mining and knowledge discovery, can extract scientific evidence, providing an auxiliary support to doctors in clinical diagnosis. Therefore, evidence-based analysis of medical evidence can not only validate the accuracy of research but also play a relevant and conclusive role in clinical decision-making.

According to our study, patient risk was divided into three levels based on calculation of their total risk through the CLS algorithm. Then, every patient was assigned a serial number and a follow-up period. Follow-up periods were based on different risk levels. Individuals comprehensively understood to provide suggestions for the follow-up period compared with considering only single or several aspects. A reasonable and sufficient recommendation was expected.

Although studies published in 2006 and later show that fewer cases progressed to CIN3+, on average, in high-risk HPV-positive women compared with studies before 2006 (26), it remains critical to identify high-risk individuals to minimize the risk of their developing high-grade precancerous lesions. CIN3+ has been shown to be predominantly attributed to persistent HPV-16 and -18 infection, in line with the present study. However, it is difficult to identify the variations in the trends of the distribution of HPV genotypes in the target population due to the effects of vaccination and other factors such as patient age.

In extant studies of HPV, patient age has not received sufficient attention. Actually, age is non-negligible as one of other factors in present and potential CIN3 cases regardless of including or excluding HPV genotype. In our study, age was found to play a key role in CIN3 risk, ranking in significance only after HPV-16 and -18 infection status. Because of the limitations of the study, we did not analyze how or why age affects the infecting HPV genotypes and CIN risk. Extant studies show that the characteristics of distribution of high-risk HPV types differ with increasing age in patients with CIN2+. For instance, HPV-16 and -18 types cause CIN more often in younger women than in older women who are affected by genotypes other than those associated with non-high-risk HPV (27, 28). The reason for the atypical age-related distribution of HPV genotypes in older women is immunological status (29). Changes in the immunological status of older women weaken their immune systems, resulting in less effective immune clearance of uncommon HPV genotypes. Persistent infection may occur for the same reason and lead to high-grade cervical lesions. In the meanwhile, the incidence of CIN2 and CIN3 in the 20–29-year age group has doubled relative to the >60-year age group (30). Approximately 50% of cervical cancer cases in older women result in non-high-risk-HPV (31). Age and immunological status ought to be fully considered when investigating the distribution of HPV genotype.

The emergence and development of HPV vaccines, from bivalent to nonavalent, as the primary prevention method, has effectively protected more and more women of the appropriate age from HPV infection with certain genotypes, with well-established safety (32). The bivalent vaccine covers the HPV genotypes 16 and 18; the quadrivalent vaccine covers the low-risk genotypes 6 and 11 that contribute to most cases of genital warts (33, 34) and the two high-risk genotypes mentioned above. In addition to all these abovementioned genotypes, high-risk HPV genotypes 31, 33, 45, 52, and 58 are the other genotypes covered by the nonavalent vaccine (35).

Extant studies describe high efficacy (>90%) of the HPV vaccine against high-grade CIN-related genotypes and persistent high-risk HPV infection, and an efficacy of 64.6% against cross-protective types (HPV-31, -33, and -45). Additionally, the HPV vaccine shows robust and long-acting clinical efficacy in terms of protection and prevention (36, 37). Due to the effects of the uptake of the HPV vaccine, changes of prevalent HPV genotypes in women of different age groups have appeared globally. However, the unequal uptake of HPV vaccination program step by step in the world has led to variations in HPV genotype among countries and regions at a given time. Studies indicate the role of the HPV vaccine in preventing the occurrence of CIN2 and CIN3 in some countries (36, 37). Although the proportion of CIN3 due to genotypes covered by the nonavalent vaccine is high in the age group of 45 years and above, it seems that older women have significantly higher risk of high-grade CIN associated with the genotypes of HPV that are not covered by the nonavalent vaccine as well as non-high-risk HPV precancerous lesions.

In the present study, we find that HPV-16, -18, and -45 are the common types leading to all stages of CIN; this is consistent with a study that considers HPV-16 and -18 as the main types of cervical squamous carcinoma and HPV-18 and -45 as the primary types of cervical adenocarcinoma (38).The HPV types at the secondary level leading to CIN did not appear to be common features, likely because of the differences in race, region, and vaccination status.

In the future, the rates of HPV-16 and -18 infection are expected to gradually decrease, especially in young women, as a result of the effectiveness of bivalent and quadrivalent HPV vaccination programs. The influence of nonavalent vaccine on other prevalent high-risk genotypes is deemed to come out in a long term for relatively late implementation and stipulated younger age group between 9 and 26. Therefore, traditional high-risk HPV types may not be predictive of CIN or lesions. Conversely, the specific HPV genotypes excluded in the nonavalent vaccination, such as -56, -66, -51, -39, -35, and -26, are expected to be predictive of CIN3 and CIN3+.

Therefore, HPV genotyping test is a valuable screening method to predict risk value and guide individual management. Clinical decision-makers should regard age as a factor, together with HPV genotype, when managing CIN3 patients (39). Overall, these findings highlight the importance of regular cervical cancer screening throughout a woman’s lifetime and tailoring management strategies based on individual risk profiles. This would allow unnecessary interventions to be minimized while ensuring early detection and treatment of precancerous lesions before they progress to invasive disease.

The HPV vaccine—an effective preventive strategy against HPV infection, related genital warts, and cervical cancer (40–42)—combined with HPV testing is expected to reduce cervical cancer rates. HPV testing has gradually become the main screening method due to its good sensitivity, and HPV self-sampling programs will be an available supplement to improve screening coverage. Although HPV self-sampling projects have been carried out only in a small number of countries, because of its advantages as a safe, simple, and private method, HPV self-sampling may have more widespread application in the future in additional countries (43). In addition, the vaccination status of girls and women should be taken into account during triage and to determine the frequency of HPV screening; these considerations should be explored in future studies (42).

## 5 Conclusion

Through in-depth study of the interactions between the risk factors for high-risk HPV, a risk factor relationship diagram structure was constructed. The risk level of patients was analyzed based on all risk factors, and a follow-up period for each risk level was formulated. According to the correlation between high-risk HPV genotypes and CIN, a lesion prediction model was constructed to predict the stage of cervical lesions within a reasonable follow-up period, provide a basis for pathological diagnosis, effectively reduce the risk of lesions, and even cancerization, and achieve primary prevention of cervical cancer.

In this study, we mined potential key risk factors, identified high-risk HPV patients, formulated follow-up periods for each risk level, and predicted cervical lesions, providing a new technological basis and ideas for related fields. Through evidence-based analysis, we demonstrated that the construction of a cervical cancer knowledge base and the structure of the risk factor relationship graph in this study play a key role in the evidence-based analysis of diseases and provide convenience and a scientific basis for evidence-based medicine. Furthermore, the findings offer time savings to doctors by enabling to assess and conduct decision making more efficiently. At the same time, the potential risk factors mined based on the structure of the risk factor map are also of significance for guiding clinical diagnosis and disease prevention. Altogether, the findings of this study can help the medical community to identify high-risk HPV patients more accurately, arrange follow-up more effectively, and improve the accuracy of cervical lesion prediction, thus providing more effective strategies for the prevention and treatment of cervical cancer.

## Data availability statement

The datasets presented in this study can be found in online repositories. The names of the repository/repositories and accession number(s) can be found in the article/supplementary material.

## Ethics statement

The studies involving humans were approved by Ethics Committee of Jilin Central General Hospital. The studies were conducted in accordance with the local legislation and institutional requirements. Written informed consent for participation was not required from the participants or the participants’ legal guardians/next of kin because the local legislation and institutional requirements.

## Author contributions

LG: Project administration, Writing – original draft, Conceptualization. YT: Data curation, Methodology, Writing – original draft. HX: Conceptualization, Investigation, Writing – original draft. LZ: Investigation, Resources, Writing – review & editing. YS: Formal analysis, Writing – review & editing, Supervision.
